# How Cardiac Output May be Described in Fontan Patients: An Approximative Equation

**DOI:** 10.1093/icvts/ivaf259

**Published:** 2025-10-27

**Authors:** Manfred Marx, Sulaima Albinni, Erwin Kitzmüller, Ina Michel-Behnke

**Affiliations:** Division of Pediatric Cardiology, Department of Pediatric and Adolescent Medicine, Medical University of Vienna, Medizinische Universität Wien, 1090 Vienna, Austria; Division of Pediatric Cardiology, Department of Pediatric and Adolescent Medicine, Medical University of Vienna, Medizinische Universität Wien, 1090 Vienna, Austria; Division of Pediatric Cardiology, Department of Pediatric and Adolescent Medicine, Medical University of Vienna, Medizinische Universität Wien, 1090 Vienna, Austria; Division of Pediatric Cardiology, Department of Pediatric and Adolescent Medicine, Medical University of Vienna, Medizinische Universität Wien, 1090 Vienna, Austria

With great interest, we have read the article by Gewillig et al^1^ offering that much clinical experience to the readers. Authors propose a formula that elucidates how flow through the Cavo-pulmonary connection (critical bottleneck), and therefore through the whole circuit, is controlled.


$$\mathrm{CO}=\frac{\Delta P}{\sum R}=\frac{\mathrm{CVP}(\mathrm{PAP})-\mathrm{LAP}}{R_{\mathrm{CPC}}+\mathrm{PVR}}+\frac{\mathrm{CVP}-\mathrm{LAP}}{R_{\mathrm{fenestration}}},$$

where CO is the cardiac output; CVP is the central venous pressure; ΔP is the pressure difference; LAP is the left atrial pressure; PAP is the pulmonary artery pressure; PVR is the pulmonary vascular resistance; RCPC is the resistance of the cavopulmonary connection; ∑R is the sum of resistances; and *R* is the resistance.

The intention of the authors is to focus on the fact that CO is mainly regulated by parameters within the formula. However, if we understand the formula as an analogue to Ohm’s law:


$$I=\frac{V}{R}∼F=\frac{\Delta P}{R},$$

where *I* is the current through a conductor; *V* is the voltage measured across the conductor, *F* is the Flow; ΔP is the pressure difference; and R is the resistance.

We are dealing more with “volume flow” (volume/time) than with real CO, as CO is strictly linked to HR (CO = stroke volume (SV) × heart rate (HR)).

According to Hagen–Poiseuille equation


$$\mathrm{VF}=\frac{\pi (p_{1}-p_{2})}{8l\eta }R^{4},$$

where VF is the volume flow; length; η is the dynamic viscosity; and *R* is the radius (vessel, capillary bed).

A change in VF can only be achieved by changing the pressure difference and/or the resistance; this means that the capillary bed plays the most important role $\left(R^{4}\right)$. This also explains why HR, especially early after creation of the Fontan circuit, has negligible or no effect on volume flow and therefore neither on congestion.

However, HR has an influence on CO in Fontan patients, in the early postoperative period, probably a negative, as any increase in HR in a volume-deprived ventricle results in a proportional decrease of VF. Later, a clear and even enhanced increase in HR relative to workload or metabolic demand regulates CO to a point at which stroke volume is falling abruptly and cardiac output is starting to plateau.^2^

To describe the blood circuit of Fontan patients in total, we would like to propose a modified equation including HR to describe CO in Fontan circulation:


$$\mathrm{CO}≅\mathrm{VF}\times \mathrm{HR}=\frac{\Delta P}{\sum R}\times \mathrm{HR}=(\frac{\mathrm{CVP}(\mathrm{PAP})-\mathrm{LAP}}{R_{\mathrm{CPC}}+\mathrm{PVR}}+\frac{\mathrm{CVP}-\mathrm{LAP}}{R_{\mathrm{fenestration}}})\times \mathrm{HR},$$

where CO is the cardiac output; VF is the volume flow; CVP is the central venous pressure; ΔP is th pressure difference; LAP is the left atrial pressure; PAP is the pulmonary artery pressure; PVR is the pulmonary vascular resistance; RCPC is the resistance of the cavo-pulmonary connection; ∑R is the sum of resistances; and *R* is the resistance.

In our opinion, this equation describes CO as approximately as possible and provides all parameters to readers necessary in functional interpretation of CO in Fontan patients.
